# Metagenomes of Red Sea Subpopulations Challenge the Use of Marker Genes and Morphology to Assess *Trichodesmium* Diversity

**DOI:** 10.3389/fmicb.2022.879970

**Published:** 2022-05-30

**Authors:** Coco Koedooder, Etai Landou, Futing Zhang, Siyuan Wang, Subhajit Basu, Ilana Berman-Frank, Yeala Shaked, Maxim Rubin-Blum

**Affiliations:** ^1^The Fredy and Nadine Herrmann Institute of Earth Sciences, Hebrew University of Jerusalem, Jerusalem, Israel; ^2^The Interuniversity Institute for Marine Sciences in Eilat, Eilat, Israel; ^3^Israel Oceanographic and Limnological Research, Haifa, Israel; ^4^Mina and Everard Goodman Faculty of Life Sciences, Bar Ilan University, Ramat Gan, Israel; ^5^Microsensor Research Group, Max Planck Institute for Marine Microbiology, Bremen, Germany; ^6^Department of Marine Biology, Leon H. Charney School of Marine Sciences, University of Haifa, Haifa, Israel

**Keywords:** *Trichodesmium*, hetR, cyanobacteria, morphotype, Red Sea, taxonomy, metagenome, biodiversity

## Abstract

*Trichodesmium* are filamentous cyanobacteria of key interest due to their ability to fix carbon and nitrogen within an oligotrophic marine environment. Their blooms consist of a dynamic assemblage of subpopulations and colony morphologies that are hypothesized to occupy unique niches. Here, we assessed the poorly studied diversity of *Trichodesmium* in the Red Sea, based on metagenome-assembled genomes (MAGs) and *hetR* gene-based phylotyping. We assembled four non-redundant MAGs from morphologically distinct *Trichodesmium* colonies (tufts, dense and thin puffs). *Trichodesmium thiebautii* (puffs) and *Trichodesmium erythraeum* (tufts) were the dominant species within these morphotypes. While subspecies diversity is present for both *T. thiebautii* and *T. erythraeum*, a single *T. thiebautii* genotype comprised both thin and dense puff morphotypes, and we hypothesize that this phenotypic variation is likely attributed to gene regulation. Additionally, we found the rare non-diazotrophic clade IV and V genotypes, related to *Trichodesmium nobis* and *Trichodesmium miru*, respectively that likely occurred as single filaments. The *hetR* gene phylogeny further indicated that the genotype in clade IV could represent the species *Trichodesmium contortum*. Importantly, we show the presence of *hetR* paralogs in *Trichodesmium*, where two copies of the *hetR* gene were present within *T. thiebautii* genomes. This may lead to the overestimation of *Trichodesmium* diversity as one of the copies misidentified *T. thiebautii* as *Trichodesmium aureum*. Taken together, our results highlight the importance of re-assessing *Trichodesmium* taxonomy while showing the ability of genomics to capture the complex diversity and distribution of *Trichodesmium* populations.

## Introduction

*Trichodesmium* is a genus of filamentous cyanobacteria known for its ability to form large visible surface blooms in tropical and subtropical regions of the ocean. Having first been described in 1770 by James Cook (Transcription of National Library of Australia, 2004), *Trichodesmium* have been extensively studied due to their ability to form blooms of large biomass supporting marine food webs through their nitrogen and carbon fixing capabilities (Dugdale et al., 1961) and subsequent relevance to biogeochemical cycles within oligotrophic marine environments (Capone et al., 1997; McKinna, 2015; Capone, 2021).

*Trichodesmium* blooms are dynamic over space and time, typically consisting of a complex assemblage of several different subpopulations and colony morphologies that are predicted to exhibit unique ecological lifestyles (Hansel et al., 2016; Gradoville et al., 2017; Eichner et al., 2019; Delmont, 2021; Held et al., 2021; Wang et al., 2021). For example, the comparison of two distinct puff-shaped morphotypes termed “dense” and “thin” colonies, isolated from the Red Sea displayed a remarkable heterogeneity in their preference to capture and center dust (Wang et al., 2021). While colony morphotypes cannot always be linked to different genotypes or species (Rouco et al., 2016; Gradoville et al., 2017) genomic information regarding *Trichodesmium* colonies in this region is lacking in comparison to studies conducted in the Atlantic and Pacific Oceans.

The diversity of *Trichodesmium* bloom-forming populations was first assessed *via* colony morphology (Janson et al., 1995). With the advent of molecular techniques, the use of single-marker genes represented a more accurate and consistent phylogenetic technique (McManus and Katz, 2009). Marker genes that have been used to assess *Trichodesmium* diversity include the 16S rRNA, *hetR*, a regulatory gene for the development of heterocysts, and *nifH*, which encodes the nitrogenase iron protein. The *hetR* gene is considered a good marker to assess *Trichodesmium* diversity as it is more variable (10%) in comparison to genetic markers such as 16S ribosomal RNA (2–3%) or *nifH* (2%; Orcutt et al., 2002; Lundgren et al., 2005).

Phylogenetic analysis using *hetR* as a marker gene separates *Trichodesmium* into four distinct clades: clade I (*Trichodesmium thiebautii*, *Trichodesmium hildebrandtii*, *Trichodesmium tenue*, and *Trichodesmium pelagicum*), clade II (*Trichodesmium aureum*), clade III (*Trichodesmium erythraeum*, *Trichodesmium havanum*), and clade IV (*Trichodesmium contortum*, *Trichodesmium tenue*; Lundgren et al., 2005; Hynes et al., 2012). Recently, a fifth clade (clade V) consisting of the non-diazotrophic *Trichodesmium miru* was proposed by Delmont (2021). Currently, genomes of clades I, III, (and V) are available, including that of the culturable clade III lineage *Trichodesmium erythraeum* IMS101 (Walworth et al., 2015).

To better understand the taxonomic diversity of *Trichodesmium* in the Red Sea, we isolated colonies from the Gulf of Aqaba and compared the resulting metagenome-assembled genomes (MAGs) to those of other *Trichodesmium* populations from the Indian, Pacific and Atlantic Oceans. We confirmed the presence of these genomes in the Red Sea based on amplicon sequencing of the *hetR* gene, and, in light of our findings, evaluated the ability of this marker gene to capture *Trichodesmium* diversity.

## Materials and Methods

### *Trichodesmium* Sampling and Extraction

**Figure 1:**
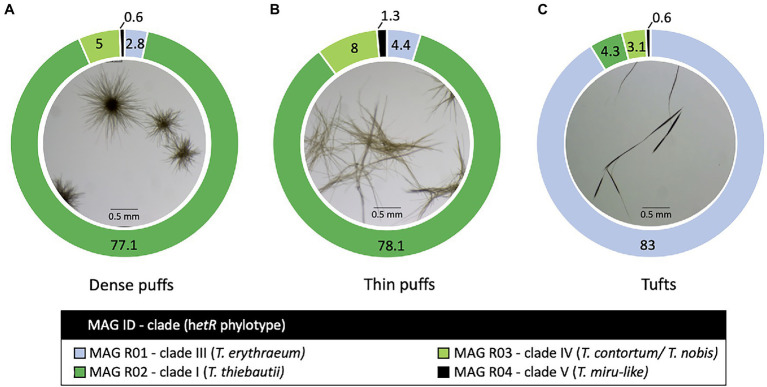
*Trichodesmium* colony morphotypes and their relative read abundance (%) in the Red Sea. Both **(A)** dense and **(B)** thin puff-shaped morphotypes, primarily consisted of *Trichodesmium thiebautii* genotype (dark green; MAG R02), while tuft-shaped morphotypes **(C)** were dominated by *Trichodesmium erythraeum* (light blue; MAG R01). All morphotypes contained low read abundance of non-diazotrophic MAG R03 (light green; MAG R03) and MAG R04 (black; MAG R04) genotypes, clustering within clade IV and V respectively.

To assess the *Trichodesmium* population of the Red Sea, *Trichodesmium* colonies were handpicked and separated into three distinct morphotypes (~100–200 colonies each) throughout the winter bloom (November 2020) using a 100 μm phytoplankton net at 20 m depth in the Gulf of Aqaba (Eilat, Israel; 29.56°N, 34.95°E; Figure 1). Colonies were washed three times in 0.2 μm filtered seawater before being filtered on a 0.2 μm PCC filter using a vacuum pump. Filters were frozen in liquid N_2_ and kept at −80°C until analysis.

DNA was extracted from three *Trichodesmium* samples using the DNAeasy Plant Mini Kit (Qiagen) following the manufacturer’s instructions. Metagenomic libraries were prepared at HyLabs (Rehovot, Israel) and sequenced with 30–60 million 2 × 150 bp reads on Illumina HiSeq 3000 at GENEWIZ (Leipzig, Germany). The metagenomic samples can be found under the accession numbers SRR17940154-6.

### Metagenomic Analysis

*Trichodesmium* MAGs were binned from assemblies based on the raw reads from this study (PRJNA804487) and those from the previously published metagenomes of *Trichodesmium* from the Pacific (PRJNA435427; PRJNA358796) and Atlantic (PRJNA330990) Oceans (Frischkorn et al., 2017, 2018a; Gradoville et al., 2017). Metagenomes were assembled, annotated, and binned using the ATLAS (v2) pipeline (Kieser et al., 2020; Supplementary R Markdown File). Briefly, raw sequences underwent quality control through the BBTools suite (Bushnell, 2014; Bushnell et al., 2017) and were assembled using metaSPAdes (Nurk et al., 2017; k-mer lengths: 21, 33, 55, 99, and 121 bp). MAGs were binned from each sample using MetaBAT 2 (Kang et al., 2019), MaxBin 2.0 (Wu et al., 2016), and VAMB (Nissen et al., 2021). The completeness and redundancy of each bin were assessed using CheckM (Parks et al., 2015). A non-redundant set of bins was produced using DAS Tool (Sieber et al., 2018) and dRep (Olm et al., 2017) based on an average nucleotide identity (ANI) cutoff of 97.5%. MAGs were taxonomically characterized using the genome taxonomy database tool kit GTDB-tk (Parks et al., 2018). Genes were predicted using Prodigal (Hyatt et al., 2010). We refer to the Red-Sea MAGs from this study as MAG R and MAGs based on other studies as MAG T. The genome of the cultured strain *Trichodesmium erythraeum* IMS101 and five TARA Oceans MAGs, including two novel non-diazotrophic genomes (Delmont, 2021), were incorporated in the follow-up analysis. The ANI values for each MAG were analyzed using ANIb module in pyANI (Pritchard et al., 2015).

### Amplicon Sequencing of the *hetR* Gene

*Trichodesmium* samples (tuft and puff morphotypes) for amplicon sequencing of the *hetR* were collected in the Gulf of Aqaba (same location as above) during several blooms (2013–2019). DNA was extracted using the phenol-chloroform method (Massana et al., 1997). Partial *hetR* gene sequences (~355 bp) were amplified using the forward primer (hetrR_50F), 5′-ATTGAACCYA AACGGGTT-3′ and reverse primer (hetR_381R), 5′-CGCTTAATATGTY CTGYCAAAGCTT-3′, which were deduced from conserved regions of a *Trichodesmium hetR* nucleotide sequence alignment. 2 × 250 bp reads were sequenced on Illumina MiSeq, following library preparation at HyLabs (Rehovot, Israel). The *hetR* amplicon samples can be found under the accession numbers SAMN25885796-803. Sequences were merged, denoised, and called into amplicon sequence variants (ASVs) using DADA2 in Qiime2 (Callahan et al., 2016; Bolyen et al., 2019). The ASVs were clustered into five OTUs at 98% similarity for further downstream analyses using the VSEARCH consensus taxonomy classifier (Rognes et al., 2016).

### *Trichodesmium* Phylogeny

**Figure 2:**
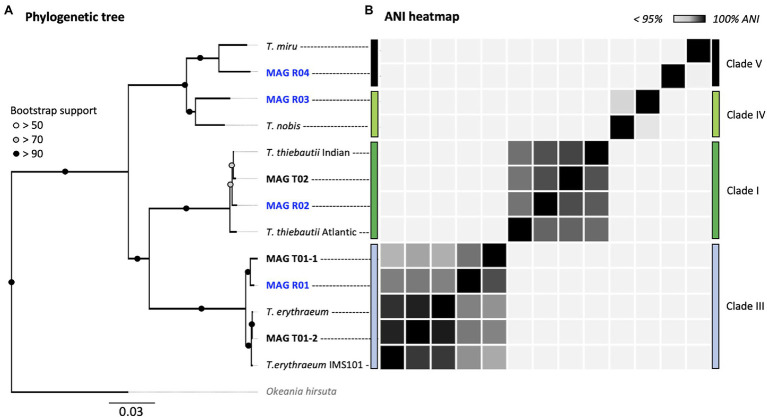
Multi-locus (251 HMMs) phylogenomic tree **(A)** and ANI heatmap **(B)** of *Trichodesmium* MAGs. All MAGs assembled in this study are marked in bold. Red Sea MAGs (our samples) are marked in blue. The phylogeny includes five MAGs from the TARA Oceans dataset and the laboratory culture *Trichodesmium erythraeum* IMS101. The tree was rooted at *Okeania hirsuta* (gray). The accession numbers for each MAG can be found in Supplementary Tables 1, 2, and their ANI values in Supplementary Tables 3a,b.

We constructed multi-locus and single marker gene (*hetR*) phylogenies of *Trichodesmium*. We used the following genomes: the four *Trichodesmium* MAGs from this study, five *Trichodesmium* MAGs from the TARA Oceans dataset (Delmont, 2021) and that of *Trichodesmium erythraeum* IMS101 PRJNA318 (Walworth et al., 2015). The phylogenomic tree was constructed from a concatenated gene-alignment of a 251 single-copy gene-set hidden Markov Models (HMMs) for Cyanobacteria using GToTree (v.1.16.12; default settings; Lee, 2019). The aligned protein sequences were refined using Gblocks (0.91b; default settings) to eliminate poorly aligned positions and divergent regions (Castresana, 2000). A tree was subsequently constructed from the cleaned alignment using IQtree2 (v2.1.3) which implements ModelFinder (v1) to estimate the best-fit model (Q.plant+F + I + G4; Kalyaanamoorthy et al., 2017; Minh et al., 2020). Shimodaira–Hasegawa approximate likelihood-ratio test (SH-aLRT) and ultrafast bootstrap approximation (UFBoot) branch support values were estimated from 1,000 bootstraps. The tree was visualized using FigTree (v1.4.4; Figure 2) and rooted with *Okeania hirsuta* (GCA_003838225; Moss et al., 2018) as an outgroup.

**Figure 3:**
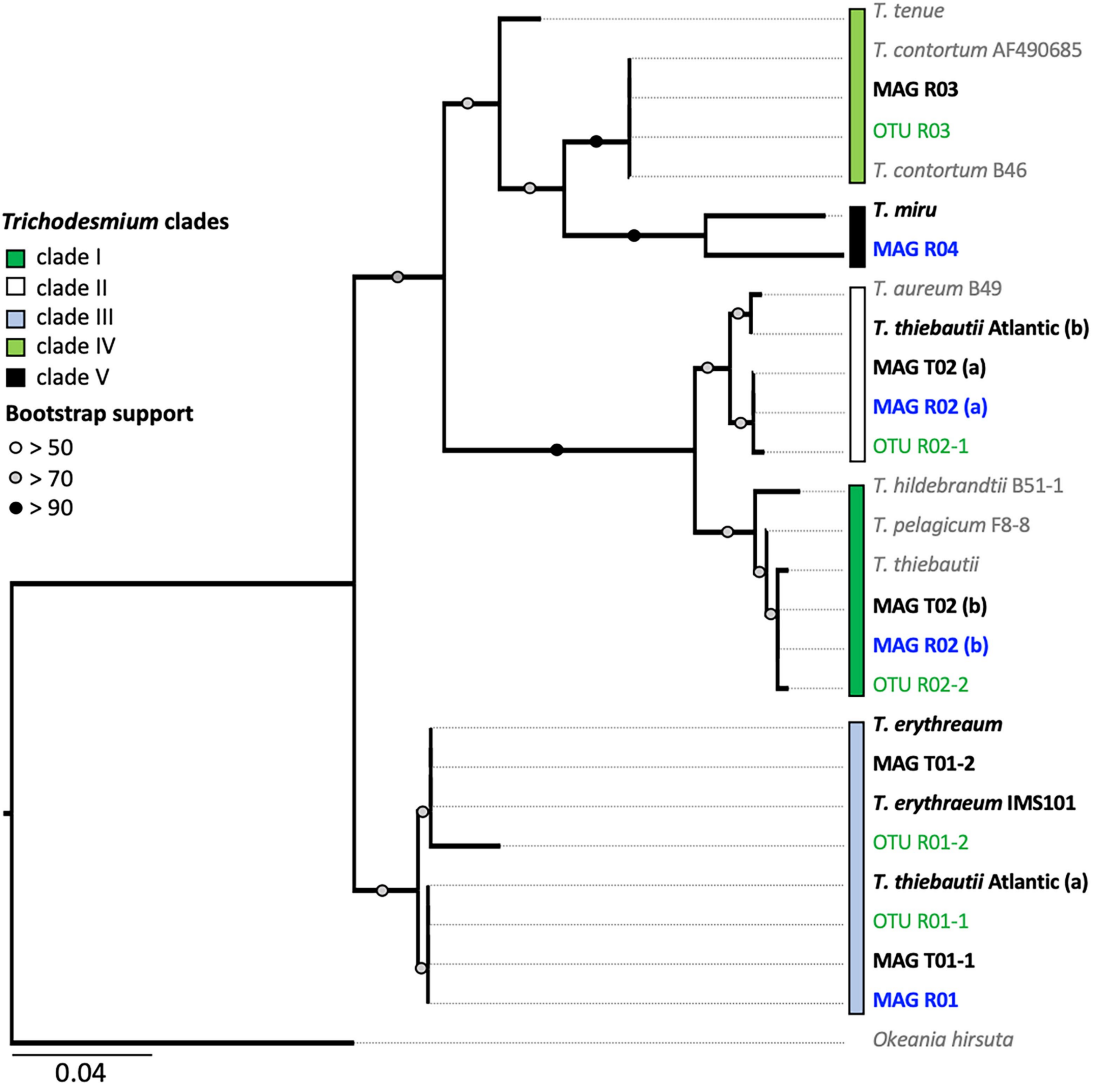
*Trichodesmium hetR* phylogeny. The tree is based on the alignment of 312 nucleotide sequences. Bold text indicates the *hetR* sequences from metagenome-assembled genomes (MAGs; Red Sea sequences are marked in blue). Amplicon *hetR* sequences are shown in green. The five proposed *Trichodemium* clades are shown with colored boxes. Note that clades IV and V are polyphyletic and are therefore not completely resolved. MAGs R02 and T03 contain two *hetR* sequences, clustering within clades I and II, respectively. The average percentage identity (ANI) matrix between the *hetR* gene sequences can be found in Supplementary Table 4.

The *hetR* marker gene was identified in *Trichodesmium* MAGs using the Rapid Annotation using Subsystem Technology (RAST v2.0; Aziz et al., 2008). The *hetR* sequence and the neighboring genes cluster were identified by searching for the HetR amino acid sequence (Tery_1921; Q93CE9) using BLAST on the SEED (v2.0) server (Aziz et al., 2012; Overbeek et al., 2013). Coverage of the raw reads from the *hetR*-containing contig was further assessed in IGV (v2.12.3; Robinson et al., 2011). The marker gene sequences from each *Trichodesmium* MAG were aligned with previously published ones (Janson et al., 1999; Orcutt et al., 2002; Hynes et al., 2012) and those from our Red Sea *hetR* amplicon sequencing using the Multiple Alignment Fast Fourier Transformation (MAFFT v7.490; L-INS-i) software (Katoh et al., 2002, 2005, 2019). All the *hetR* sequences used in this study can be found in Supplementary Table 5. This alignment was further cleaned using GBlocks (v0.91b; default settings; Castresana, 2000). A phylogenetic tree rooted at the sequence of *Okeania hirsuta*, was constructed using IQtree2 (v2.1.3) for *hetR* (best-fit model TPM3 + F + I) and visualized using FigTree (v1.4.4; Figure 3). Similarly, the phylogeny of the *rbcL* gene from *Trichodesmium* MAGs was also assessed (best-fit model TN + F + G4) and visualized in IQtree2 (Supplementary Figure 1).

## Results and Discussion

Four non-redundant *Trichodesmium* MAGs were assembled from the Red Sea (Figure 1; Supplementary Table 1). Briefly, MAG R01 (*T. erythraeum*) and MAG R04 (*T. miru*-like) were assembled from the tuft sample. MAG R02 (*T. thiebautii*) was curated from dense-puff samples, while MAG R03 (*T. nobis-like*) was from long puffs. The MAGs were of high quality, being 90% complete and with less than 5% redundancy, apart from MAG R04 which was 86.29% complete. To provide a wider framework of *Trichodesmium* diversity, we assembled three non-redundant *Trichodesmium* MAGs from the Pacific and the Atlantic Ocean, based on previously published raw data (Frischkorn et al., 2017, 2018b; Gradoville et al., 2017). These included two high-quality MAGs T01-2 and T02, as well as the lower quality MAG T01-1 (Supplementary Table 2).

Our results suggest that *T. erythraeum* and *T. thiebautii* are the dominant *Trichodesmium* lineages in the Red Sea, where MAG R01 clustered with *T. erythraeum* (97.3% ANI), and MAG R02 with *T. thiebautii* (97.7–98.3% ANI; Figure 2; Supplementary Table 3). The dominance of these lineages in the Red Sea could be confirmed from *hetR* gene phylotyping of *Trichodesmium* populations across several seasons (Supplementary Figure 2). Tuft colonies were primarily composed of *T. erythraeum* (~83% read abundance; Figure 1C; Orcutt et al., 2002).

Both thin and dense puff-shaped colonies were dominated by *T. thiebautii* (~78% read abundance; Supplementary Table 1; Figures 1A,B). This finding is similar to another genomic study where radial (dense-like) and non-radial puff morphologies were linked to the same genotype of *T. thiebautii* (Gradoville et al., 2017). Puff-shaped *Trichodesmium* colonies have the unique capability to interact, capture, and concentrate dust at the colony’s core (Rubin et al., 2011) which can allow colonies to obtain limiting nutrients from the marine environment, such as iron or phosphorous (Basu and Shaked, 2018; Basu et al., 2019). In the field, thin puffs isolated from the Red Sea were shown to be more interactive in comparison to dense puffs in the capturing and concentrating of dust at the colony’s core, while exhibiting pronounced gliding motility (Wang et al., 2021). Our findings highlight that these morphological variations do not appear to be explained genomically and that other yet uncharacterized factors are at play. We hypothesize that the observed variations in puff morphologies concerning the centering of dust (Kessler et al., 2019; Wang et al., 2021) are due to differences in gene regulation in response to yet unknown environmental conditions which may include nutrient limitation or grazing. In a laboratory setting, single filaments of *T. erythraeum* IMS101 clustered into the puff and tuft-shaped colonies when subjected to iron or phosphorous limitation (Tzubari et al., 2018). Nonetheless, *Trichodesmium* displays functional variability and heterogeneity at a single-colony level that remains largely unexplained (Eichner et al., 2019; Held et al., 2021) and subsequently the ability to predict whether a colony will center dust still requires ongoing exploration.

Our data revealed subspecies diversity for both *T. thiebautii* and *T. erythraeum* (Figure 2). *Trichodesmium thiebautii* MAGs appeared to cluster according to broad geographical patterns as MAG R02 was more similar to that of *T. thiebautii* populations isolated from the Indian Ocean (98.3% ANI), than to the Atlantic *T. thiebautii* MAG (97.7% ANI). This concurs with the previous findings (Delmont, 2021). We also found genomic diversity within *T. erythraeum* as revealed from two distinct *T. erythraeum* MAGs from the Pacific Ocean (96.6% ANI). MAG T01-1 was highly similar to that of *T. erythraeum* isolated from the Red Sea (97.6% ANI; MAG R01), whereas MAG T01-2 was closely related to *T. erythraeum* IMS101 (99.4% ANI), which was isolated from the Atlantic Ocean (Prufert-Bebout et al., 1993). The *hetR* gene phylotyping of *Trichodesmium* populations in the Red Sea indicates the occurrence of an additional *T. erythraeum* subspecies similar to that of MAG T01-2 (Figure 3). We were, however, unable to assemble its genome or link it to a specific morphotype. It is still unclear if these two *T. erythraeum* subspecies within the Red Sea and the Pacific Ocean occupy distinct ecological niches.

We detected two potentially non-diazotrophic species within the Red Sea with minor read abundance in all three samples (Supplementary Table 1). Both genotypes could not be linked to a specific morphotype, and it is plausible that they either interleaved with *T. thiebautii* puffs or *T. erythraeum* tufts, or existed as single filaments. The low coverage reflected a previous estimate of the non-diazotrophic *Trichodesmium* of the Red Sea (Delmont, 2021). Whereas MAG R03 clustered within clade IV, which includes the non-diazotrophic *T. nobis* (95.6% ANI), and MAG R04 clustered within clade V containing the non-diazotrophic *T. miru* (94.6% ANI), both MAGs are on the threshold of being a distinct species within clade IV and V, respectively. We, therefore, turned to the single-marker gene *hetR* to further differentiate these MAGs, as *hetR* phylogeny captures a larger diversity of *Trichodesmium* than is possible through the limited number of *Trichodesmium* genomes currently available. The *hetR* sequences, but not the genomes, are available for *T. pelagicum* (AF490696.1), *T. hildebrandtii* (AF490679.1) in clade I, *T. aureum* (AF490680.1) in clade II, and *T. contortum* (AF013031.1), *T. tenue* (AF013033.1) in clade IV and *T. miru* in clade V.

We show that *T. nobis* and *T. contortum* are closely related, potentially representing the same non-diazotrophic *Trichodesmium* species. Given that *T. nobis* has not been described morphologically, and the genome of *T. contortum* has not been sequenced, both can be linked through the phylogeny of marker genes. Whereas the previously published genome of *T. nobis* did not contain the *hetR* gene (Delmont, 2021), the clade IV MAG R03 did, and its *hetR* gene sequence was 99.78% similar to that of *T. contortum* (Figure 3; Supplementary Table 4). Complementing our findings, morphological descriptions of *T. contortum* depict the species as single spiral-shaped trichomes, rather than colonies, that are sporadically present in low abundance (<1% of total *Trichodesmium* biomass) within samples (Janson et al., 1995; Letelier and Karl, 1996; Orcutt et al., 2002). MAG R03 lacked the nitrogenase gene cluster, confirming that this clade is non-diazotrophic.

**Figure 4:**
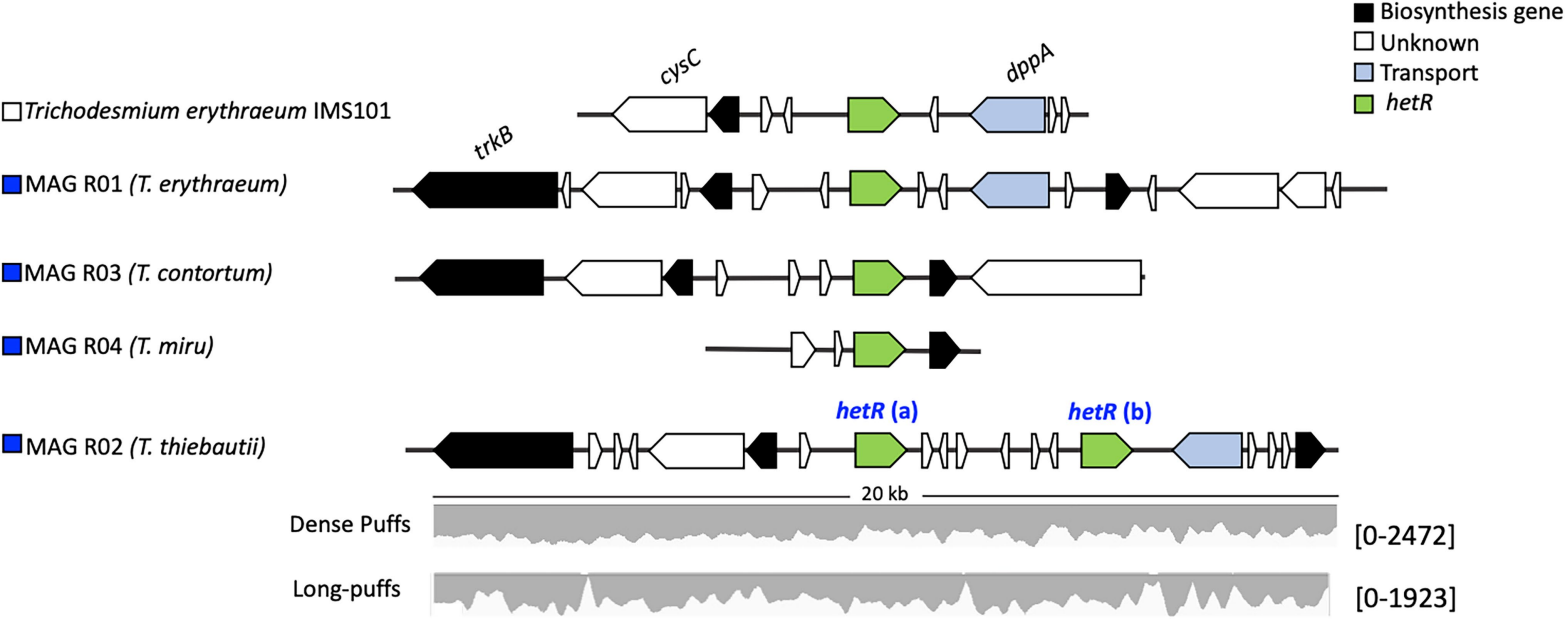
The *hetR* gene clusters in *Trichodesmium* sp. The synteny of the *hetR* genes (green) and the neighboring genes of the cluster is conserved between the *Trichodesmium* MAGs and *Trichodesmium erythraeum* IMS101. MAG R02 (*Trichodesmium thiebautii*) contains two *hetR* genes. Raw read counts, listed within the brackets, were mapped to the *hetR* gene cluster of MAG R02 from both the dense and long puff samples.

Our results indicate that *hetR* phylotyping can overestimate the diversity within *Trichodesmium* populations, in particular regarding clade II. Intriguingly, both *T. thiebautii* MAGs contained two distinct *hetR* copies (906 bp), which had 28 (MAG R02) and 29 bp (MAG T02) single nucleotide polymorphisms within a sequence. Further inspection of the two copies showed that they were encoded in close vicinity of each other and that the synteny is highly conserved across all the *Trichodesmium* genomes (Figure 4). Metagenomic reads of MAG T02 mapped uniformly to both *hetR* copies in both the dense (50.53 and 49.47% *hetR* reads) and long-puff samples (49.21 and 50.21% *hetR* reads), indicating that they likely originate from a single population DNA. The putative pseudogenes between the two *hetR* genes did not match any known viral sequence, isolate spacer, or metagenomic spacer within the Integrated Microbial Genome/Virus Repository (IMG/VR v3; 10^−5^ cutoff value; Roux et al., 2021). Therefore, we suspect that these *hetR* genes are likely paralogs, although it is still unclear how *T. thiebautii* benefits from retaining both *hetR* sequences. Each *hetR* sequence was placed into a distinct phylogenetic clade, where one copy grouped with the *hetR* sequence of *T. aureum* (clade II), and the other one was placed within clade I, together with *T. thiebautii*, *T. pelagicum*, and *T. hildebrandtii* (Figure 3).

Amplicon sequencing of the *hetR* gene showed highly similar abundances of *T. thiebautii* and *T. aureum* during most sampling days. Some fluctuations in the relative read abundance were observed (Supplementary Figure 2). These findings can hint at the occurrence of *T. aureum* in the Red Sea. Yet, the presence of *T. aureum*-like *hetR* sequence as a paralog in a single population of *T. thiebautii* can lead to similar observations. *Trichodesmium aureum* has previously been described based on cloning and Sanger sequencing of the *hetR* gene whereas its morphology has been described poorly (Lundgren et al., 2005; Hynes et al., 2012). Therefore, past distinctions made between *T. thiebautii* and *T. aureum* may reflect the morphological diversity within the same genotype or species. We note that the Atlantic *T. thiebautii* contained one *hetR* paralog that clustered with the *hetR* sequence of clade IV (*T. erythraeum*), yet we cannot exclude a binning artifact in this case, as the sequences did not belong to a single scaffold.

## Conclusion and Future Perspectives

Morphotype metagenomics enabled us to better capture the diversity that is present within *Trichodesmium* populations, while revisiting past attempts to differentiate between colonies (Scornavacca et al., 2020; Smith and Hahn, 2021). We were able to reconstruct the genomes of four different *Trichodesmium* species isolated from the Red Sea, that were subsequently assigned to four different *Trichodesmium* clades including the more elusive non-diazotrophic clades IV and V. Single-colony, rather than bulk population analysis, may help address whether these non-diazotrophic lineages occur as single free-floating filaments, from colonies of yet unknown morphology or are included within the single *T. erythraeum* puff or *T. thiebautii* puff colonies. We further show that *hetR* gene-based phylotyping could overestimate *Trichodesmium* diversity, highlighting the importance of re-assessing past attempts at phylogeny. Other marker genes such as full-length 16S rRNA as suggested by Delmont (2021) or the single-copy cyanobacterial marker *rbcL* (Singh et al., 2015) may assess the phylogenetic diversity of *Trichodesmium* (Supplementary Figure 1), although further verification will be required. Overall, the phylogenetic and functional diversity of *Trichodesmium* in the world’s oceans is still poorly understood to date. Thus, genomics in combination with gene expression and physiology, especially as single-colony approaches, will remain an important avenue for future exploration of these key species.

## Data Availability Statement

The four Trichodesmium genomes, the raw metagenomic sequences and the hetR amplicon sequencing data presented in this study are deposited under the BioProject ID number PRJNA804487.

## Author Contributions

CK, YS, and MR-B conceived the study. CK, FZ, and SW collected and extracted the metagenomic samples. Under guidance of MR-B, CK analyzed the metagenomic data. EL and SB collected samples for *hetR* amplicon sequencing. Under the guidance of IB-F, EL extracted and analyzed the amplicon sequencing data. The manuscript was compiled and written by CK with the help of MR-B. YS, MR-B, IB-F, and EL provided feedback and guidance during the writing process. All authors contributed to the article and approved the submitted version.

## Funding

This work was funded by the Israel Science Foundation (260/21) and the Israel-United States Bi-National Science Foundation (2020041) to YS. This work was also financially supported in part by the Schulich Marine Studies Initiative to IB-F. MR-B acknowledges the support of the Israel Science Foundation (913/19), the United States-Israel Binational Science Foundation (2019055), and the Israel Ministry of Science and Technology (1126). FZ thanks the PBC Fellowship Program for Outstanding Chinese and Indian Post-Doctoral Fellows.

## Conflict of Interest

The authors declare that the research was conducted in the absence of any commercial or financial relationships that could be construed as a potential conflict of interest.

## Publisher’s Note

All claims expressed in this article are solely those of the authors and do not necessarily represent those of their affiliated organizations, or those of the publisher, the editors and the reviewers. Any product that may be evaluated in this article, or claim that may be made by its manufacturer, is not guaranteed or endorsed by the publisher.

## References

[ref1] Aziz R. K. Bartels D. Best A. DeJongh M. Disz T. Edwards R. A.. (2008). The RAST server: rapid annotations using subsystems technology. BMC Genomics 9, 1–15. doi: 10.1186/1471-2164-9-75, PMID: 18261238 PMC2265698

[ref2] Aziz R. K. Devoid S. Disz T. Edwards R. A. Henry C. S. Olsen G. J.. (2012). SEED servers: high-performance access to the SEED genomes, annotations, and metabolic models. PLoS One 7:e48053. doi: 10.1371/journal.pone.0048053, PMID: 23110173 PMC3480482

[ref3] Basu S. Gledhill M. de Beer D. Matondkar S. G. P. Shaked Y. (2019). Colonies of marine cyanobacteria *Trichodesmium* interact with associated bacteria to acquire iron from dust. Commun. Biol. 2, 284–288. doi: 10.1038/s42003-019-0534-z, PMID: 31396564 PMC6677733

[ref4] Basu S. Shaked Y. (2018). Mineral iron utilization by natural and cultured *Trichodesmium* and associated bacteria. Limnol. Oceanogr. 63, 2307–2320. doi: 10.1002/lno.10939

[ref5] Bolyen E. Rideout J. R. Dillon M. R. Bokulich N. A. Abnet C. C. Al-Ghalith G. A.. (2019). Reproducible, interactive, scalable and extensible microbiome data science using QIIME 2. Nat. Biotechnol. 37, 852–857. doi: 10.1038/s41587-019-0209-9, PMID: 31341288 PMC7015180

[ref6] Bushnell B. (2014). BBTools software package. Available at: https://sourceforge.net/projects/bbmap/ (Accessed December 16, 2021).

[ref7] Bushnell B. Rood J. Singer E. (2017). BBMerge—accurate paired shotgun read merging via overlap. PLoS One 12:e0185056. doi: 10.1371/journal.pone.0185056, PMID: 29073143 PMC5657622

[ref8] Callahan B. J. McMurdie P. J. Rosen M. J. Han A. W. Johnson A. J. A. Holmes S. P. (2016). DADA2: high-resolution sample inference from Illumina amplicon data. Nat. Methods 13, 581–583. doi: 10.1038/nmeth.3869, PMID: 27214047 PMC4927377

[ref9] Capone D. G. (2021). Coming full circle on diazotrophy in the marine cyanobacterium *Trichodesmium*. Proc. Natl. Acad. Sci. U. S. A. 118:e2117967118. doi: 10.1073/pnas.2117967118, PMID: 34785598 PMC8617427

[ref10] Capone D. G. Zehr J. P. Paerl H. W. Bergman B. Carpenter E. J. (1997). *Trichodesmium*, a globally significant marine cyanobacterium. Science 276, 1221–1229. doi: 10.1126/science.276.5316.1221

[ref11] Castresana J. (2000). Selection of conserved blocks from multiple alignments for their use in phylogenetic analysis. Mol. Biol. Evol. 17, 540–552. doi: 10.1093/oxfordjournals.molbev.A026334, PMID: 10742046

[ref12] Delmont T. O. (2021). Discovery of nondiazotrophic *Trichodesmium* species abundant and widespread in the open ocean. Proc. Natl. Acad. Sci. U. S. A. 118:e2112355118. doi: 10.1073/PNAS.2112355118, PMID: 34750267 PMC8609553

[ref13] Dugdale R. C. Menzel D. W. Ryther J. H. (1961). Nitrogen fixation in the Sargasso Sea. Deep-Sea Res. 7, 297–300. doi: 10.1016/0146-6313(61)90051-X

[ref14] Eichner M. Basu S. Gledhill M. de Beer D. Shaked Y. (2019). Hydrogen dynamics in *Trichodesmium* colonies and their potential role in mineral iron acquisition. Front. Microbiol. 10:1565. doi: 10.3389/fmicb.2019.01565, PMID: 31354665 PMC6636555

[ref15] Frischkorn K. R. Haley S. T. Dyhrman S. T. (2018a). Coordinated gene expression between *Trichodesmium* and its microbiome over day-night cycles in the north pacific subtropical gyre. ISME J. 12, 997–1007. doi: 10.1038/s41396-017-0041-5, PMID: 29382945 PMC5864210

[ref16] Frischkorn K. R. Krupke A. Guieu C. Louis J. Rouco M. Estrada A. E. S.. (2018b). *Trichodesmium* physiological ecology and phosphate reduction in the western tropical South Pacific. Biogeosciences 15, 5761–5778. doi: 10.5194/BG-15-5761-2018

[ref17] Frischkorn K. R. Rouco M. van Mooy B. A. S. Dyhrman S. T. (2017). Epibionts dominate metabolic functional potential of *Trichodesmium* colonies from the oligotrophic ocean. ISME J. 11, 2090–2101. doi: 10.1038/ismej.2017.74, PMID: 28534879 PMC5563961

[ref18] Gradoville M. R. Crump B. C. Letelier R. M. Church M. J. White A. E. (2017). Microbiome of *Trichodesmium* colonies from the north pacific subtropical gyre. Front. Microbiol. 8:1122. doi: 10.3389/fmicb.2017.01122, PMID: 28729854 PMC5498550

[ref19] Hansel C. M. Buchwald C. Diaz J. M. Ossolinski J. E. Dyhrman S. T. van Mooy B. A. S.. (2016). Dynamics of extracellular superoxide production by *Trichodesmium* colonies from the Sargasso Sea. Limnol. Oceanogr. 61, 1188–1200. doi: 10.1002/lno.10266

[ref20] Held N. A. Sutherland K. M. Webb E. A. McIlvin M. R. Cohen N. R. Devaux A. J.. (2021). Mechanisms and heterogeneity of in situ mineral processing by the marine nitrogen fixer *Trichodesmium* revealed by single-colony metaproteomics. ISME Commun. 1, 1–9. doi: 10.1038/s43705-021-00034-y PMC9723768 36739337

[ref21] Hyatt D. Chen G. L. LoCascio P. F. Land M. L. Larimer F. W. Hauser L. J. (2010). Prodigal: prokaryotic gene recognition and translation initiation site identification. BMC Bioinformatics 11, 1–11. doi: 10.1186/1471-2105-11-119, PMID: 20211023 PMC2848648

[ref22] Hynes A. M. Webb E. A. Doney S. C. Waterbury J. B. (2012). Comparison of cultured *Trichodesmium* (Cyanophyceae) with species characterized from the field. J. Phycol. 48, 196–210. doi: 10.1111/J.1529-8817.2011.01096.X, PMID: 27009664

[ref23] Janson S. Bergman B. Carpenter E. J. Giovannoni S. J. Vergin K. (1999). Genetic analysis of natural populations of the marine diazotrophic cyanobacterium *Trichodesmium*. FEMS Microbiol. Ecol. 30, 57–65. doi: 10.1111/J.1574-6941.1999.TB00635.X

[ref24] Janson S. Siddiqui P. J. A. Walsby A. E. Romans K. M. Carpenter E. J. Bergman B. (1995). Cytomorphological of the planktonic diazotrophic cyanobacteria *Trichodesmium* spp. from the Indian Ocaen and Caribbean and Sargasso seas. J. Phycol. 31, 463–477. doi: 10.1111/J.0022-3646.1995.00463.X

[ref25] Kalyaanamoorthy S. Minh B. Wong T. von Haeseler A. Jermiin L. (2017). ModelFinder: fast model selection for accurate phylogenetic estimates. Nat. Methods 14, 587–589. doi: 10.1038/nmeth.4285, PMID: 28481363 PMC5453245

[ref26] Kang D. D. Li F. Kirton E. Thomas A. Egan R. An H.. (2019). MetaBAT 2: an adaptive binning algorithm for robust and efficient genome reconstruction from metagenome assemblies. PeerJ 7:e7359. doi: 10.7717/PEERJ.7359, PMID: 31388474 PMC6662567

[ref27] Katoh K. Kuma K. I. Toh H. Miyata T. (2005). MAFFT version 5: improvement in accuracy of multiple sequence alignment. Nucleic Acids Res. 33, 511–518. doi: 10.1093/nar/gki198, PMID: 15661851 PMC548345

[ref28] Katoh K. Misawa K. Kuma K. I. Miyata T. (2002). MAFFT: a novel method for rapid multiple sequence alignment based on fast Fourier transform. Nucleic Acids Res. 30, 3059–3066. doi: 10.1093/nar/gkf436, PMID: 12136088 PMC135756

[ref29] Katoh K. Rozewicki J. Yamada K. D. (2019). MAFFT online service: multiple sequence alignment, interactive sequence choice and visualization. Brief. Bioinform. 20, 1160–1166. doi: 10.1093/bib/bbx108, PMID: 28968734 PMC6781576

[ref30] Kessler N. Armoza-Zvuloni R. Wang S. Basu S. Weber P. K. Stuart R. K.. (2019). Selective collection of iron-rich dust particles by natural *Trichodesmium* colonies. ISME J. 14, 91–103. doi: 10.1038/s41396-019-0505-x, PMID: 31551530 PMC6908701

[ref31] Kieser S. Brown J. Zdobnov E. M. Trajkovski M. McCue L. A. (2020). ATLAS: a snakemake workflow for assembly, annotation, and genomic binning of metagenome sequence data. BMC Bioinformatics 21:257. doi: 10.1186/S12859-020-03585-4, PMID: 32571209 PMC7310028

[ref33] Lee M. D. (2019). GToTree: a user-friendly workflow for phylogenomics. Bioinformatics 35, 4162–4164. doi: 10.1093/bioinformatics/btz188, PMID: 30865266 PMC6792077

[ref34] Letelier R. M. Karl D. M. (1996). Role of *Trichodesmium* spp. in the productivity of the subtropical north pacific ocean. Mar. Ecol. Prog. Ser. 133, 263–273. doi: 10.3354/MEPS133263

[ref35] Lundgren P. Janson S. Jonasson S. Singer A. Bergman B. (2005). Unveiling of novel radiations within *Trichodesmium* cluster by hetR gene sequence analysis. Appl. Environ. Microbiol. 71, 190–196. doi: 10.1128/AEM.71.1.190-196.2005, PMID: 15640187 PMC544273

[ref36] Massana R. Murray A. E. Preston C. M. DeLong E. F. (1997). Vertical distribution and phylogenetic characterization of marine planktonic Archaea in the Santa Barbara channel. Appl. Environ. Microbiol. 63, 50–56. doi: 10.1128/aem.63.1.50-56.1997, PMID: 8979338 PMC168301

[ref37] McKinna L. I. W. (2015). Three decades of ocean-color remote-sensing *Trichodesmium* spp. in the world’s oceans: a review. Prog. Oceanogr. 131, 177–199. doi: 10.1016/j.pocean.2014.12.013

[ref38] McManus G. B. Katz L. A. (2009). Molecular and morphological methods for identifying plankton: what makes a successful marriage? J. Plankton Res. 31, 1119–1129. doi: 10.1093/plankt/fbp061

[ref39] Minh B. Q. Schmidt H. A. Chernomor O. Schrempf D. Woodhams M. D. von Haeseler A.. (2020). IQ-TREE 2: new models and efficient methods for phylogenetic inference in the genomic era. Mol. Biol. Evol. 37, 1530–1534. doi: 10.1093/molbev/msaa015, PMID: 32011700 PMC7182206

[ref40] Moss N. A. Leão T. Rankin M. R. McCullough T. M. Qu P. Korobeynikov A.. (2018). Ketoreductase domain dysfunction expands chemodiversity: malyngamide biosynthesis in the cyanobacterium *Okeania hirsuta*. ACS Chem. Biol. 13, 3385–3395. doi: 10.1021/ACSCHEMBIO.8B00910, PMID: 30444349 PMC6470004

[ref41] Nissen J. N. Johansen J. Allesøe R. L. Sønderby C. K. Armenteros J. J. A. Grønbech C. H.. (2021). Improved metagenome binning and assembly using deep variational autoencoders. Nat. Biotechnol. 39, 555–560. doi: 10.1038/S41587-020-00777-4, PMID: 33398153

[ref42] Nurk S. Meleshko D. Korobeynikov A. Pevzner P. A. (2017). metaSPAdes: a new versatile metagenomic assembler. Genome Res. 27, 824–834. doi: 10.1101/GR.213959.116, PMID: 28298430 PMC5411777

[ref43] Olm M. R. Brown C. T. Brooks B. Banfield J. F. (2017). dRep: a tool for fast and accurate genomic comparisons that enables improved genome recovery from metagenomes through de-replication. ISME J. 11, 2864–2868. doi: 10.1038/ismej.2017.126, PMID: 28742071 PMC5702732

[ref44] Orcutt K. M. Rasmussen U. Webb E. A. Waterbury J. B. Gundersen K. Bergman B. (2002). Characterization of *Trichodesmium* spp. by genetic techniques. Appl. Environ. Microbiol. 68, 2236–2245. doi: 10.1128/AEM.68.5.2236-2245.2002, PMID: 11976093 PMC127538

[ref45] Overbeek R. Olson R. Pusch G. D. Olsen G. J. Davis J. J. Disz T.. (2013). The SEED and the rapid annotation of microbial genomes using subsystems technology (RAST). Nucleic Acids Res. 42, D206–D214. doi: 10.1093/nar/gkt1226, PMID: 24293654 PMC3965101

[ref46] Parks D. H. Chuvochina M. Waite D. W. Rinke C. Skarshewski A. Chaumeil P. A.. (2018). A standardized bacterial taxonomy based on genome phylogeny substantially revises the tree of life. Nat. Biotechnol. 36, 996–1004. doi: 10.1038/nbt.4229, PMID: 30148503

[ref47] Parks D. H. Imelfort M. Skennerton C. T. Hugenholtz P. Tyson G. W. (2015). CheckM: assessing the quality of microbial genomes recovered from isolates, single cells, and metagenomes. Genome Res. 25, 1043–1055. doi: 10.1101/GR.186072.114, PMID: 25977477 PMC4484387

[ref48] Pritchard L. Glover R. H. Humphris S. Elphinstone J. G. Toth I. K. (2015). Genomics and taxonomy in diagnostics for food security: soft-rotting enterobacterial plant pathogens. Anal. Methods 8, 12–24. doi: 10.1039/C5AY02550H

[ref49] Prufert-Bebout L. Paerl H. W. Lassen C. (1993). Growth, nitrogen fixation, and spectral attenuation in cultivated *Trichodesmium* species. Appl. Environ. Microbiol. 59, 1367–1375. doi: 10.1128/AEM.59.5.1367-1375.1993, PMID: 16348931 PMC182091

[ref50] Robinson J. T. Thorvaldsdóttir H. Winckler W. Guttman M. Lander E. S. Getz G.. (2011). Integrative genomics viewer. Nat. Biotechnol. 29, 24–26. doi: 10.1038/NBT.1754, PMID: 21221095 PMC3346182

[ref51] Rognes T. Flouri T. Nichols B. Quince C. Mahé F. (2016). VSEARCH: a versatile open source tool for metagenomics. PeerJ 4:e2584. doi: 10.7717/PEERJ.2584, PMID: 27781170 PMC5075697

[ref52] Rouco M. Haley S. T. Dyhrman S. T. (2016). Microbial diversity within the *Trichodesmium* holobiont. Environ. Microbiol. 18, 5151–5160. doi: 10.1111/1462-2920.13513, PMID: 27581522

[ref53] Roux S. Páez-Espino D. Chen I. M. A. Palaniappan K. Ratner A. Chu K.. (2021). IMG/VR v3: an integrated ecological and evolutionary framework for interrogating genomes of uncultivated viruses. Nucleic Acids Res. 49, D764–D775. doi: 10.1093/NAR/GKAA946, PMID: 33137183 PMC7778971

[ref54] Rubin M. Berman-Frank I. Shaked Y. (2011). Dust-and mineral-iron utilization by the marine dinitrogen-fixer *Trichodesmium*. Nat. Geosci. 4, 529–534. doi: 10.1038/ngeo1181

[ref55] Scornavacca C. Delsuc F. Galtier N. (eds.) (2020). Phylogenetics in the Genomic Era. Available at: https://hal.inria.fr/PGE/ (Accessed December 14, 2021).

[ref56] Sieber C. M. K. Probst A. J. Sharrar A. Thomas B. C. Hess M. Tringe S. G.. (2018). Recovery of genomes from metagenomes via a dereplication, aggregation and scoring strategy. Nat. Microbiol. 3, 836–843. doi: 10.1038/s41564-018-0171-1, PMID: 29807988 PMC6786971

[ref57] Singh P. Fatma A. Mishra A. K. (2015). Molecular phylogeny and evogenomics of heterocystous cyanobacteria using rbcl gene sequence data. Ann. Microbiol. 65, 799–807. doi: 10.1007/S13213-014-0920-1

[ref58] Smith M. L. Hahn M. W. (2021). New approaches for inferring phylogenies in the presence of paralogs. Trends Genet. 37, 174–187. doi: 10.1016/J.TIG.2020.08.012, PMID: 32921510

[ref59] Transcription of National Library of Australia (2004). Cook’s Journal: Daily Entries, 22 August 1770. South Seas, 287. Available at: http://nla.gov.au/nla.cs-ss-jrnl-cook-17700822 (Accessed February 10, 2022).

[ref60] Tzubari Y. Magnezi L. Be’er A. Berman-Frank I. (2018). Iron and phosphorus deprivation induce sociality in the marine bloom-forming cyanobacterium *Trichodesmium*. ISME J. 12, 1682–1693. doi: 10.1038/s41396-018-0073-5, PMID: 29463890 PMC6018766

[ref61] Walworth N. Pfreundt U. Nelson W. C. Mincer T. Heidelberg J. F. Fu F.. (2015). Trichodesmium genome maintains abundant, widespread noncoding DNA in situ, despite oligotrophic lifestyle. Proc. Natl. Acad. Sci. U. S. A. 112, 4251–4256. doi: 10.1073/PNAS.1422332112, PMID: 25831533 PMC4394263

[ref62] Wang S. Koedooder C. Zhang F. Kessler N. Eichner M. Shi D.. (2021). Colonies of the marine cyanobacterium *Trichodesmium* optimize dust utilization by selective collection and retention of nutrient-rich particles. iScience 25:103587. doi: 10.1016/J.ISCI.2021.103587, PMID: 35005537 PMC8718973

[ref63] Wu Y. W. Simmons B. A. Singer S. W. (2016). MaxBin 2.0: an automated binning algorithm to recover genomes from multiple metagenomic datasets. Bioinformatics 32, 605–607. doi: 10.1093/BIOINFORMATICS/BTV638, PMID: 26515820

