# Supplementary material for: Metagenomes of Red Sea Subpopulations Challenge the Use of Marker Genes and Morphology to Assess Trichodesmium Diversity
Source: Front Microbiol. 2022 May 30;13:879970. doi: 10.3389/fmicb.2022.879970 (PMC9189399; doi:10.3389/fmicb.2022.879970)
Supplement: Supplementary file 2 [file Data_Sheet_1.PDF]

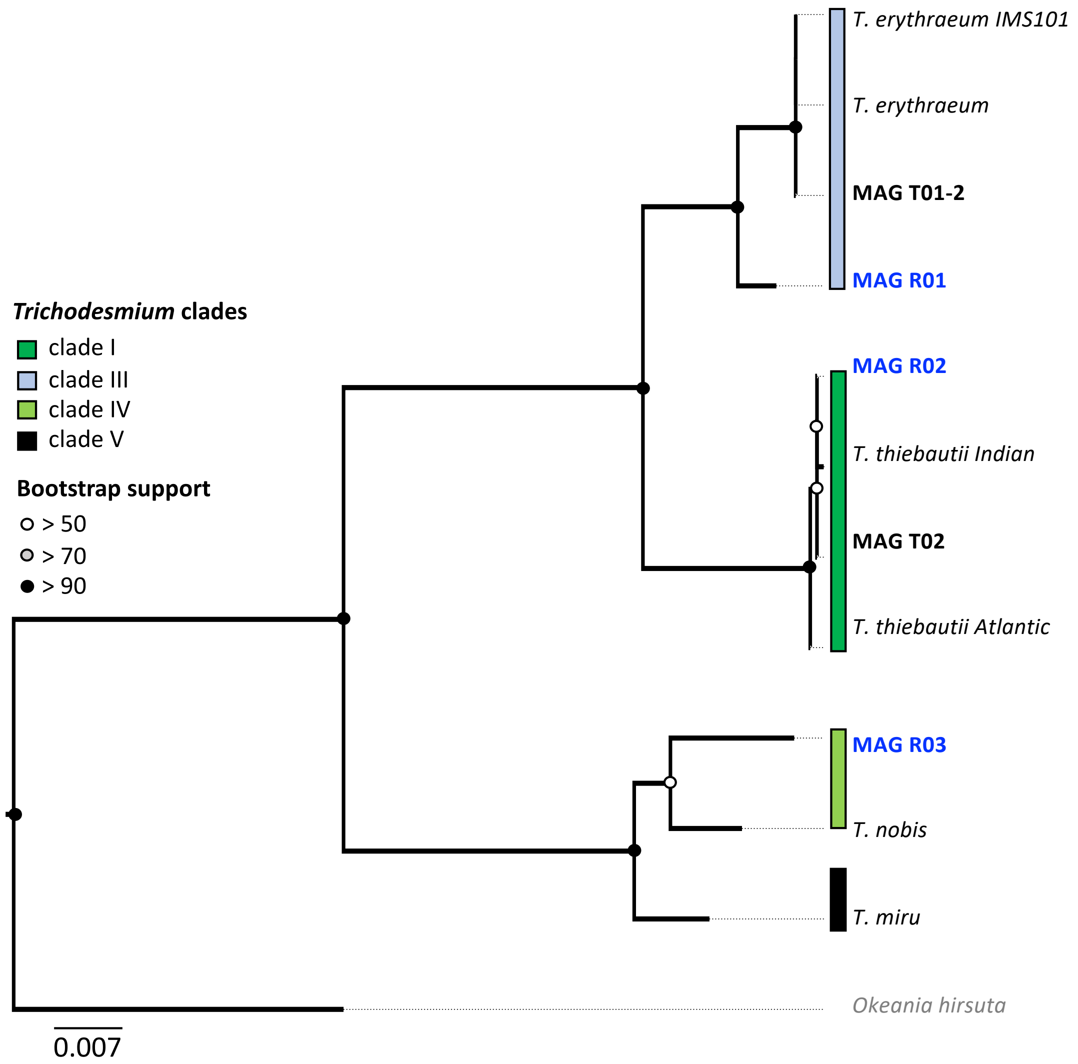

Supplementary Figure 1. *Trichodesmium rbcL phylogeny. Bold text indicates the hetR sequences from MAGs (Red Sea sequences are marked in blue). The five proposed Trichodemium clades are shown with colored boxes and is congruent with phylogenomics, although an rbcL gene was missing in MAG T01-1. Nonetheless, rbcL may serve as an alternative to address the diversity of Trichodesmium populations by amplicon sequencing, although will need to be verified experimentally.*

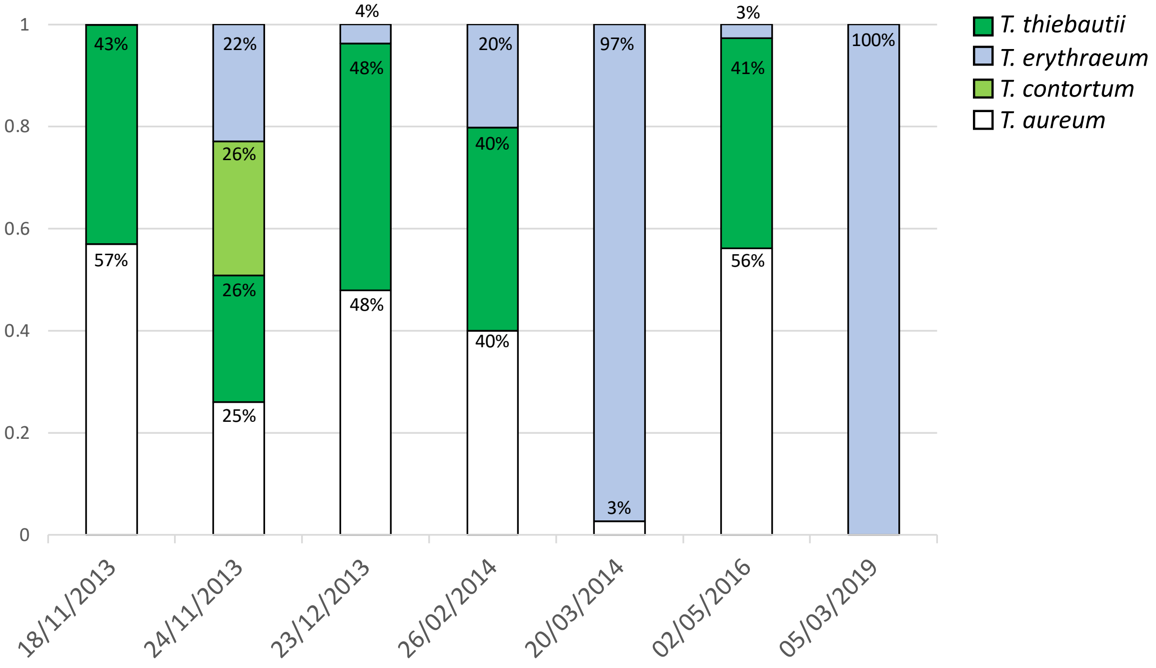

Supplementary Figure 2. *Displaying the relative abundance of hetR sequences from (puff and tuft) Trichodesmium colonies isolated from the Red Sea. Note that for most sampling days T. thiebautii and T. aureum are in similar proportions to each other. The presence of T. aureum is likely an artifact and instead represents a paralog hetR sequence present in T. thiebautii, thereby effectively over-estimating Trichodesmium diversity within the sample.*
